# Supplementary material for: Cord Blood Adductomics Reveals Oxidative Stress Exposure Pathways of Bronchopulmonary Dysplasia
Source: Antioxidants (Basel). 2024 Apr 20;13(4):494. doi: 10.3390/antiox13040494 (PMC11047351; doi:10.3390/antiox13040494)
Supplement: Supplementary file 1 [file antioxidants-13-00494-s001.zip › Supplemental Table S1_antioxidants_4_19_2024.pdf]

Table S1. Putative HSA-Cys^{34} adducts in cord and peripheral blood plasma.

Adduct	Putative Annotation	Known / Unknown	Elemental composition of added mass	m/z	Added Mass (Da)	HRMS Retention Time (min)	QQQ Retention Time (min)	PAR (× 1,000)[a]	Concentration (pmol/ mg HSA)[b]	References
A001	-Lys from C-terminus	Known	-Lys	769.59	-128.10	38.48	6.80	0.107	0.010	[37, 49]
A002	Cys^{34}→Gly	Known	$-CH_2S$	796.96	-45.99	27.50	4.75	0.405	0.040	[14, 30, 31, 33, 34, 35, 37]
A003	Cys^{34}→Dehydroalanine	Known	$-SH_2$	800.96	-33.99	28.40	5.20	0.014	0.001	[14, 30, 31, 33]
A004	Cys^{34}→ Oxoalanine or formylglycine	Known	$-SH_2$, +O	806.29	-18.00	27.20	5.60	0.101	0.010	[14, 30, 31,33,34,35]
A005	T3 dimer	Known	$+C_{114}H_{172}N_{27}O_{30}S$	811.42	2431.25	30.37	5.25	627.152	61.394	[14, 28, 30, 31,33, 32]
A006	Unmodified T3	Known	+H	812.29	1.01	28.60	5.16	568.851	55.687	[14, 28, 30, 31,32, 33, 34]
A007	CH_2 crosslink	Known	$+CH_2O$, $-H_2O$	815.95	12.00	25.61	3.10	1.067	0.104	[34, 35]
A008	Cys^{34}-Gln cross-link (monooxidation), Cys^{34} Sulfinamide	Known	$-H_2$, +O	816.65	13.98	29.68	5.20	24.267	2.376	[14, 28, 30, 31,32,33, 34,35]
A009	Methylation (not at Cys34)	Known	$+CH_3$	816.96	15.02	29.10	5.32	19.704	1.929	[14, 28, 30, 31,32, 33,34,35]
A010	*S*-Sodiation	Known	+Na	819.62	22.99	26.70	5.16	29.622	2.900	[28, 33, 34, 35,]
A011	*S*-Cyanylation	Known	+CN	820.62	26.00	28.80	5.20	1.680	0.164	[14, 30, 31, 32, 34, 35]
A012	dehydrated form of Cys^{34} sulfinic acid plus methylation (not Cys^{34})	Known	+CHO	821.96	28.99	30.10	4.90	0.020	0.002	[32]
A013	dehydrated form of Cys^{34} sulfonic acid (trioxidation)	Known	$-H_2$, $+O_2$	822.62	30.97	30.29	5.00	1.287	0.126	[32]
A014	Cys^{34} sulfinic acid (dioxidation)	Known	$+HO_2$	822.95	32.99	29.68	4.42	1.073	0.105	[14, 28, 30, 31, 32, 33,34,35]
A015	K adduct of T3	Known	-H+K	824.94	38.96	28.51	5.16	1.108	0.109	[33, 35]
A016	Ethylene oxide adduct	Known	$+C_2H_5O$	826.97	45.03	27.90	5.10	0.241	0.024	[14, 28, 30]

A017	*S*-Methylthiolation	Known	$+CH_3S$	827.62	47.00	28.80	4.99	0.070	0.007	[28, 30, 31,33, 34]
A018	*S*-Methylthiolation	Known	$+CH_3S$	827.62	47.00	28.80	4.99	0.055	0.005	[28, 30, 31,33, 34]
A019	*S*–(O)–O–CH_3	Known	$+CH_3O_2$	827.63	47.01	27.46	5.10	0.633	0.062	[28, 30, 31]
A020	Cys^{34} sulfinic acid plus methylation (not Cys^{34})	Known	$+HO_2$, $+CH_2$	827.96	47.00	30.10	5.20	1.229	0.120	[30, 32]
A021	*S*-Methylthiolation	Known	$+CH_3S$	827.99	47.00	-	-	0.319	0.031	-
A022	Cys^{34} sulfonic acid (trioxidation)	Known	$+HO_3$	828.29	48.99	29.93	4.49	1.038	0.102	[14, 28, 30, 31, 32, 33, 34, 35]
A023	Acrylonitrile adduct	Known	$+C_3H_4N$	829.97	54.04	28.90	5.00	3.856	0.378	[14, 28, 30]
A024	Na adduct of Cys^{34} sulfinic acid	Known	$+HO_2$+Na-H	830.62	54.98	27.25	4.50	0.330	0.032	[14]
A025	Putative *S*-addition of acrolein	Known	$+C_3H_5O$	830.96	57.03	25.90	3.20	0.184	0.018	[34, 35]
A026	Methylisocyanate adduct	Known	$+C_2H_4NO$	831.30	58.03	27.50	5.00	1.702	0.167	[14, 30]
A027	*S*-Addition of SO_2	Known	$+HO_2S$	833.64	64.97	27.80	5.10	0.087	0.008	[30, 31]
A028	*S*-Addition of crotonaldehyde	Known	$+C_4H_7O$	835.64	71.05	28.20	5.28	0.636	0.062	[14, 28, 30, 31, 34, 35]
A029	*S*-Phenylation	Known	$+C_6H_5$	837.63	77.04	27.40	4.80	0.084	0.008	[30, 31]
A030	*S*-Addition of tiglic aldehyde	Known	$+C_5H_9O$	840.31	85.06	27.70	4.90	0.132	0.013	[30, 31, 34]
A031	*S*-Addition of pyruvate or malonate semialdehyde	Known	$+C_3H_5O_3$	841.63	89.02	28.50	4.90	0.571	0.056	[30, 33, 32]
A032	*S*-Addition of mercaptoacetic acid	Known	$+C_2H_3O_2S$	842.28	90.98	28.60	4.95	1.130	0.111	[14, 28, 30, 31,33, 32]
A033	*S*-Mercaptoacetamide	Known	$+C_2H_4NOS$	842.33	90.00	27.80	4.95	1.090	0.107	[14, 30]
A034	*S*-Addition of Cys (-H_2O)	Known	$+C_3H_4NOS$	845.95	102.00	27.70	4.15	2.020	0.198	[14, 28, 30, 31, 33, 32]
A035	*S*-Cys (possibly $NH_2 \rightarrow OH$, -H_2O)	Known	$+C_3H_3O_2S$	846.66	102.98	28.60	5.70	0.003	0.000	[30, 33]
A036	*S*-addition of benzaldehyde or quinone methide	Known	$+C_7H_7O$	847.64	107.05	31.58	5.50	0.100	0.010	[14, 30, 32, 33]
A037	*S*-Methylethyl-sulfonylation	Known	$+C_3H_7O_2S$	848.00	107.01	26.70	4.70	0.003	0.000	[30, 31]
A038	*S*-Addition of S_2O_3H	Known	$+HO_3S_2$	849.66	112.94	28.00	5.00	0.177	0.017	[14, 30, 31]

A039	*S*-Addition of hCys (-H_2O)	Known	+C_4H_6NOS	850.63	116.02	27.90	4.30	0.007	0.001	[14, 28, 30, 31, 33, 34, 35]
A040	*S*-Cys	Known	+$C_3H_6NO_2S$	851.96	120.01	26.36	3.57	393.568	38.528	[14, 28, 30, 31, 32, 33, 34]
A041	*S*-Addition of Cys (NH_2→OH)	Known	+$C_3H_5O_3S$	852.67	121.00	27.80	3.60	109.060	10.676	[14, 28, 30, 31, 32, 33]
A042	*S*-Addition of BDE	Known	+$C_6H_9O_3$	854.97	129.05	25.90	3.20	0.007	0.001	[30, 31]
A043	Oxindole	Known	+C_8H_6NO	855.97	132.04	29.00	3.80	0.250	0.024	[30, 33]
A044	*S*-Addition of hCys	Known	+$C_4H_8NO_2S$	856.63	134.02	27.00	3.62	23.199	2.271	[14, 28, 30, 31, 32, 33, 34]
A045	*S*-Addition of Cys, methylation	Known	+$C_4H_8NO_2S$	856.63	134.02	-	3.60	25.351	2.482	-
A046	*S*-Addition of hCys (NH_2→OH)	Known	+$C_4H_7O_3S$	857.34	135.01	27.15	3.70	1.293	0.127	[28, 30, 31]
A047	Na adduct of *S*-Cys	Known	+$C_3H_6NO_2S$+Na-H	859.28	141.99	28.71	3.57	21.324	2.087	[14, 28, 31, 33, 32]
A048	*S*-hCys plus methylation (not Cys^{34})	Known	+$C_4H_8NO_2S$+CH_2	861.35	148.03	29.30	3.80	0.992	0.097	[28, 30, 32, 33]
A049	*S*-Addition of CysGly (-H_2O)	Known	+$C_5H_7N_2O_2S$	865.01	159.02	27.50	3.60	2.651	0.260	[30, 31]
A050	*S*-(N-acetyl)Cys	Known	+$C_5H_8NO_3S$	866.01	162.02	28.30	5.40	0.673	0.066	[14, 30, 32, 33]
A051	*S*-Addition of CysGly	Known	+$C_5H_9N_2O_3S$	870.97	177.03	26.50	3.30	40.189	3.934	[14, 28, 30, 31. 32. 33, 34, 35]
A052	*S*-CysGly plus methylation (not Cys^{34})	Known	+$C_5H_9N_2O_3S$+CH_2	875.64	191.04	27.50	3.40	1.696	0.166	[30, 33, 32]
A053	Na adduct of *S*-CysGly	Known	+$C_5H_9N_2O_3S$+Na-H	878.68	199.01	26.10	3.20	1.351	0.132	[33]
A054	K Adduct of *S*-CysGly	Known	+$C_5H_9N_2O_3S$+K-H	884.05	214.98	25.36	3.10	0.124	0.012	[31, 33]
A055	*S*-Addition of GluCys	Known	+$C_8H_{13}N_2O_5S$	894.97	249.05	27.00	3.68	0.552	0.054	[13, 28, 30, 31,32, 33]
A056	*S*-Addition of GSH	Known	+$C_{10}H_{16}N_3O_6S$	913.98	306.07	26.90	3.54	0.678	0.066	[14, 28, 30, 31, 32, 33, 34, 35]
A057	Unknown (-12.96 Da), 2nd	Unknown	-	807.43	-14.56	31.17	3.90	0.091	0.009	-
A058	Unknown (-12.96 Da)	Unknown	-	807.97	-12.96	31.17	3.90	0.015	0.001	-
A059	Unknown (-10.07 Da)	Unknown	-	809.46	-8.48	-	5.00	2.883	0.282	-
A060	Unknown (8.85 Da)	Unknown	-	815.24	8.87	36.82	5.67	0.004	0.000	-
A061	Unknown (34.92 Da)	Unknown	-	823.93	34.92	27.17	4.10	0.007	0.001	-

A062	Unknown (53.11 Da)	Unknown	-	829.93	52.93	28.60	5.09	3.665	0.359	-
A063	Unknown (likely *S*-addition of an aldehyde)	Unknown	-	830.97	56.05	27.30	5.25	0.862	0.084	-
A064	Unknown (62.01 Da)	Unknown	-	832.96	62.01	26.32	3.60	0.005	0.001	-
A065	Unknown (101.06 Da)	Unknown	-	845.99	101.12	26.05	3.60	0.069	0.007	-
A066	Unknown (109.03 Da)	Unknown	-	848.63	109.03	27.30	5.11	0.012	0.001	-
A067	Unknown (111.03 Da)	Unknown	-	849.28	110.98	38.20	6.26	0.005	0.000	-
A068	Unknown (126.08 Da)	Unknown	-	854.31	126.07	27.70	4.90	0.005	0.000	-
A069	Unknown (137.06 Da)	Unknown	-	858.00	137.14	27.40	4.20	0.271	0.027	-
A070	Unknown (138.06 Da)	Unknown	-	858.34	138.15	26.39	4.20	0.160	0.016	-
A071	Unknown (143 Da)	Unknown	-	859.94	142.96	25.50	2.56	0.512	0.050	-
A072	Unknown (151.99 Da)	Unknown	-	862.95	151.99	26.08	3.20	1.013	0.099	-
A073	Unknown (+154.35 Da), 2nd	Unknown	-	863.20	152.75	31.83	5.20	0.009	0.001	-
A074	Unknown (153.05 Da)	Unknown	-	863.30	153.05	25.97	3.24	0.631	0.062	-
A075	Unknown (+155.07 Da), 2nd	Unknown	-	863.44	153.47	32.47	3.80	1.661	0.163	-
A076	Unknown (+154.35 Da)	Unknown	-	863.74	154.35	31.83	5.20	0.008	0.001	-
A077	Unknown (+155.07 Da)	Unknown	-	863.98	155.07	32.47	3.80	2.588	0.253	-
A078	Unknown (156.09 Da)	Unknown	-	864.32	156.10	34.76	5.50	0.004	0.000	-
A079	Unknown (156.96 Da)	Unknown	-	864.61	156.97	26.20	3.62	1.321	0.129	-
A080	Unknown (180.02 Da)	Unknown	-	872.26	179.93	-	3.35	1.645	0.161	-
A081	Unknown (183.02 Da)	Unknown	-	873.29	183.02	26.70	3.39	0.083	0.008	-
A082	Unknown (185.02 Da)	Unknown	-	873.96	185.02	27.22	4.95	0.010	0.001	-
A083	Unknown (192 Da)	Unknown	-	875.95	190.99	27.70	3.40	0.836	0.082	-
A084	Unknown (202.02 Da)	Unknown	-	879.63	202.02	-	4.95	0.002	0.000	-
A085	Unknown (212.32 Da)	Unknown	-	883.06	212.32	-	4.95	0.010	0.001	-
A086	Unknown (247.1 Da)	Unknown	-	894.66	247.12	26.10	3.70	0.564	0.055	-
A087	Unknown (262.1 Da)	Unknown	-	899.64	262.06	29.30	3.92	0.013	0.001	-
A088	Unknown (319.09 Da)	Unknown	-	918.65	319.09	29.20	3.14	3.038	0.297	-
A089	Unknown (+320.97 Da), 2nd	Unknown	-	918.74	319.37	30.96	3.14	2.961	0.290	-

A090	Unknown (+322.08 Da), 2nd	Unknown	-	919.11	320.48	30.52	3.70	0.013	0.001	-
A091	Unknown (+320.97 Da)	Unknown	-	919.28	320.97	30.52	3.70	0.007	0.001	-
A092	Unknown (322.08 Da)	Unknown	-	919.65	322.08	30.52	3.70	0.006	0.001	-
A093	Unknown (+340.08 Da), 2nd	Unknown	-	925.11	338.48	30.63	3.78	0.004	0.000	-
A094	Unknown (+340.08 Da)	Unknown	-	925.65	340.08	30.63	3.78	0.004	0.000	-
A095	Unknown (346.14 Da)	Unknown	-	927.67	346.15	27.10	2.90	0.091	0.009	-
A096	Unknown (351.07 Da)	Unknown	-	929.31	351.07	32.20	5.20	0.037	0.004	-
A097	Unknown (360.19 Da)	Unknown	-	932.35	360.19	25.37	3.05	0.284	0.028	-
A098	Unknown (388.2 Da)	Unknown	-	941.69	388.21	25.30	3.02	0.194	0.019	-
A099	Unknown (461.2 Da)	Unknown	-	966.02	461.20	25.40	3.05	1.263	0.124	-
A100	Unknown (+476 Da)	Unknown	-	970.69	475.21	28.10	3.13	0.048	0.005	-
A101	Unknown (+489 Da)	Unknown	-	975.04	488.25	25.33	2.98	0.349	0.034	-
A102	Unknown (+495.21 Da, M49,OS39)	Unknown	-	977.35	495.19	26.96	3.52	0.941	0.092	-
A103	Unknown (+509.21 Da)	Unknown	-	982.03	509.23	25.30	2.95	0.016	0.002	-
A104	Unknown (+545.22 Da), 2nd	Unknown	-	993.49	543.62	27.63	2.50	0.440	0.043	-
A105	Unknown (+545.22 Da)	Unknown	-	994.03	545.22	27.63	2.50	0.475	0.047	-

[a] Peak-Area Ratio = Adduct peak area / Housekeeping peptide peak area. [b] Estimated adduct concentrations.
