# Supplementary material for: Cord Blood Adductomics Reveals Oxidative Stress Exposure Pathways of Bronchopulmonary Dysplasia
Source: Antioxidants (Basel). 2024 Apr 20;13(4):494. doi: 10.3390/antiox13040494 (PMC11047351; doi:10.3390/antiox13040494)
Supplement: Supplementary file 1 [file antioxidants-13-00494-s001.zip › Supplemental Table S2_antioxidants_4_19_2024.pdf]

Table S2. Median concentrations according to health condition and collection timepoint.

Adduct #	Cord Blood			Peripheral Blood					
	Full-Term	No BPD	BPD	No BPD			BPD		
				1-week	1-month	36-weeks	1-week	1-month	36-weeks
A001	0.09 [0.02-0.26]	0.34 [0.1-0.8]	0.38 [0.12-1.01]	0.25 [0.06, 0.75]	0.05 [0.04, 0.12]	0.1 [0.03, 0.24]	0.12 [0.04, 0.41]	0.16 [0.06, 0.36]	0.14 [0.03, 0.42]
A002	0.76 [0.41-1.19]	0.64 [0.21-1.04]	0.45 [0.26-0.96]	1.15 [0.42, 1.96]	0.74 [0.42, 1.96]	0.86 [0.45, 1.94]	0.77 [0.23, 1.21]	1.57 [0.46, 2.12]	0.31 [0.16, 1.38]
A003	0.02 [0-0.06]	0.03 [0.01-0.12]	0.02 [0.01-0.1]	0.03 [0.02, 0.14]	0.03 [0.02, 0.3]	0.04 [0.01, 0.11]	0.04 [0.01, 0.14]	0.03 [0.01, 0.2]	0.08 [0.02, 0.33]
A004	0.21 [0.1-0.4]	0.14 [0.05-0.33]	0.14 [0.04-0.46]	0.96 [0.22, 4.34]	0.53 [0.21, 69.91]	0.33 [0.17, 0.67]	0.62 [0.15, 1.3]	0.36 [0.13, 0.68]	1.06 [0.08, 231.29]
A005	674.54 [416.09-948.26]	709.68 [425.35-1010.34]	777.2 [511.17-1019.32]	1070.6 [659.35, 1508.62]	731.54 [463.08, 1169.27]	312.6 [20.26, 1064.32]	763.07 [378.73, 945.27]	562.43 [206.24, 1218.92]	503.27 [255.71, 1162.24]
A006	704.42 [481.41-1068.38]	822.28 [474.3-1047.25]	781.68 [537.59-1072.16]	1182.25 [568.15, 1481.2]	682.14 [445.94, 1130.06]	699.34 [355.34, 1032.5]	729.6 [385.17, 1138.08]	584.71 [308.45, 1114.39]	475.04 [275.05, 1127.61]
A007	1.59 [0.81-2.68]	2.06 [0.87-3]	1.78 [1.14-3.05]	2.38 [1.61, 5.88]	1.81 [0.75, 4.74]	2.34 [1.63, 4.12]	1.66 [0.69, 3.22]	1.78 [0.49, 3.48]	1.12 [0.65, 1.69]
A008	28.71 [19-50.67]	33.89 [18.04-50.18]	37.61 [21.94-60.06]	111.36 [53.45, 151.01]	37.82 [26.44, 87.26]	45.12 [21, 71.84]	45.37 [26.3, 104.18]	37.12 [17.71, 86.41]	25.41 [12.65, 64.71]
A009	26.32 [13.31-37.89]	29.95 [15.51-42.6]	33.77 [17.71-50.13]	76.24 [38.84, 121.65]	30.68 [21.42, 68.47]	28.38 [13.43, 69.3]	37.94 [23.92, 65.56]	21.09 [14.35, 79.22]	22.04 [9.84, 44.03]
A010	32.74 [16.72-51.99]	32.75 [17.39-53.38]	35.04 [20.33-57.64]	161.11 [71.97, 283.28]	114.84 [46, 196.76]	48.54 [25.94, 143.49]	86.24 [38.11, 149.18]	62.06 [29.48, 146.9]	74.64 [14.06, 173.95]
A011	2.37 [1.34-4.03]	2.44 [1.31-4.04]	2.57 [1.37-4.49]	3.59 [2.07, 8.57]	3.85 [2.1, 6.79]	2.17 [1.03, 7.02]	2.78 [1.44, 5.2]	3.14 [1.51, 7.14]	2.71 [0.79, 5.37]
A012	0.04 [0.01-0.14]	0.03 [0-0.12]	0.05 [0-0.17]	0.06 [0.02, 0.37]	0.16 [0.02, 0.45]	0.06 [0.04, 0.12]	0.03 [0.01, 0.35]	0.11 [0.03, 0.25]	0.05 [0.02, 0.33]
A013	1.87 [1.29-3.26]	2.1 [0.98-3.7]	1.95 [1.11-3.59]	3.25 [0.68, 6.14]	4.2 [1.57, 8.23]	3.18 [2.48, 7.25]	2.96 [1.38, 6.12]	2.34 [1.24, 7.07]	2.16 [1.15, 7.39]
A014	1.6 [0.91-2.73]	1.55 [0.88-2.58]	1.71 [0.9-2.57]	2.81 [1.13, 7.78]	2.28 [1.14, 6.57]	4.1 [1.64, 5.42]	2.16 [1.25, 5.37]	2.05 [0.84, 5.66]	1.7 [0.69, 7.69]
A015	1.42 [0.66-2.35]	1.14 [0.61-2.74]	1.42 [0.83-2.96]	7.71 [4.28, 15.91]	6.24 [2.19, 8.9]	1.51 [0.18, 3.04]	3.74 [1.13, 6.31]	3.35 [1.14, 7.54]	5.17 [0.48, 13.36]
A016	0.29 [0.16-0.56]	0.3 [0.14-0.6]	0.37 [0.19-0.59]	1.88 [0.5, 3.8]	1.33 [0.83, 2.52]	1.23 [0.46, 1.9]	1.04 [0.49, 1.56]	1.57 [0.17, 2.72]	1.05 [0.42, 1.75]
A017	0.35 [0.2-0.89]	0.39 [0.16-0.85]	0.4 [0.17-0.83]	1.15 [0.53, 2.7]	1.45 [0.74, 2.55]	1.21 [0.39, 2.58]	0.87 [0.48, 2.19]	1.19 [0.49, 2.08]	0.65 [0.4, 2.03]
A018	0.07 [0.03-0.3]	0.06 [0.02-0.15]	0.11 [0.04-0.23]	0.15 [0.02, 0.64]	0.4 [0.2, 0.69]	0.14 [0.08, 0.51]	0.19 [0.04, 0.59]	0.35 [0.1, 0.61]	0.31 [0.11, 0.64]
A019	1.48 [0.53-2.57]	0.55 [0.25-0.97]	0.59 [0.29-1.06]	1.77 [0.9, 3.38]	3 [1.85, 5.3]	1.97 [1.4, 4.02]	2.67 [1.33, 3.21]	3.7 [1.67, 5.92]	1.31 [0.81, 5.09]
A020	2.49 [1.31-4.58]	1.24 [0.34-2.24]	0.85 [0.35-1.72]	3.17 [1.04, 5.89]	6.28 [2.81, 8.35]	4.75 [3.39, 6.14]	2.61 [1.49, 4.27]	4.38 [1.7, 11.68]	2.85 [0.78, 6.15]
A021	0.1 [0.04-0.39]	0.06 [0.02-0.17]	0.12 [0.04-0.29]	0.15 [0.02, 0.64]	0.4 [0.2, 0.69]	0.14 [0.08, 0.51]	0.19 [0.04, 0.59]	0.35 [0.1, 0.61]	0.31 [0.11, 0.64]
A022	2.43 [1.01-4.67]	0.79 [0.36-2.02]	0.98 [0.37-2.05]	2.86 [0.78, 4.06]	3.77 [2.33, 9]	2.97 [1.97, 4.27]	2.57 [0.89, 3.33]	3.53 [1.7, 5.58]	2.73 [0.99, 6.19]
A023	4.84 [2.08-8.91]	5.66 [2.38-10.63]	5.58 [2.88-10.1]	16.22 [6.47, 24.7]	8.38 [6.45, 13.34]	8.14 [3.17, 13.91]	4.5 [2.6, 12.02]	5.28 [2.95, 11.1]	5.86 [2.13, 14.1]
A024	0.44 [0.25-0.78]	0.4 [0.15-0.78]	0.37 [0.24-0.65]	1.63 [0.31, 3.23]	1.16 [0.8, 2.22]	0.66 [0.49, 0.94]	0.93 [0.36, 1.95]	0.97 [0.28, 2.54]	0.7 [0.23, 1.87]

**A025**	0.27 [0.13-0.48]	0.22 [0.12-0.44]	0.29 [0.12-0.61]	1.06 [0.23, 2.46]	0.46 [0.23, 1.04]	0.79 [0.44, 1.47]	0.41 [0.19, 1.49]	0.69 [0.23, 1.62]	0.42 [0.19, 0.82]
**A026**	2.3 [1.02-3.27]	2.41 [1.15-3.67]	2.34 [1.45-4.09]	9.8 [2.55, 13.11]	4.98 [2.88, 7.91]	2.96 [1.1, 6.93]	4.9 [1.62, 10.43]	2.99 [1.4, 6.79]	3.27 [0.94, 5.7]
**A027**	0.12 [0.05-0.21]	0.11 [0.05-0.23]	0.16 [0.06-0.26]	0.39 [0.18, 0.74]	0.44 [0.14, 0.68]	0.18 [0.09, 0.88]	0.46 [0.27, 1.49]	0.3 [0.14, 0.67]	0.52 [0.06, 1.45]
**A028**	0.72 [0.39-1.13]	1.11 [0.42-1.72]	1.15 [0.47-1.64]	3.26 [1.75, 7.55]	2.89 [1.91, 4.91]	2.21 [0.44, 4.55]	1.64 [0.76, 4.29]	2.18 [0.41, 4.38]	1.1 [0.21, 2.09]
**A029**	0.11 [0.06-0.24]	0.11 [0.04-0.28]	0.11 [0.05-0.2]	0.79 [0.51, 1.37]	0.28 [0.13, 0.88]	0.49 [0.12, 0.95]	0.58 [0.18, 0.76]	0.3 [0.1, 0.72]	0.11 [0.04, 0.86]
**A030**	0.15 [0.06-0.31]	0.16 [0.07-0.41]	0.15 [0.07-0.29]	0.43 [0.14, 1.16]	0.68 [0.27, 1.25]	0.38 [0.21, 0.52]	0.51 [0.19, 1.3]	0.52 [0.22, 1.52]	0.31 [0.24, 0.95]
**A031**	0.75 [0.32-1.32]	0.5 [0.22-1.04]	0.54 [0.28-0.98]	6.06 [2.13, 7.72]	5.36 [2.58, 8.1]	3.05 [1.24, 3.63]	5.21 [1.91, 7.03]	3.85 [0.67, 7.49]	1.05 [0.47, 4.94]
**A032**	1.21 [0.6-2.24]	0.97 [0.4-1.92]	0.98 [0.47-1.93]	8.87 [3.22, 18.17]	6.45 [4, 12.96]	4 [2.34, 7.31]	7.18 [4.3, 15.21]	4.77 [1.81, 12.37]	2.26 [1.54, 7.92]
**A033**	1.07 [0.62-2.14]	1.16 [0.46-1.95]	1.11 [0.55-2.03]	8.01 [4.07, 19.8]	7.29 [3.43, 15.41]	3.5 [1.88, 8.39]	7.09 [3.13, 15.63]	7.01 [1.89, 14.05]	2.11 [0.87, 10.79]
**A034**	2.65 [1.24-6.43]	2.46 [1.29-5.3]	2.28 [1.23-4.64]	4.77 [1.48, 22.37]	9.37 [6.1, 20.58]	8.12 [4.52, 10.62]	6.16 [2.75, 11.07]	6.12 [2.86, 12.56]	3.67 [1.67, 16.87]
**A035**	0 [0-0]	0 [0-0.01]	0 [0-0.01]	0.02 [0.01, 0.03]	0.01 [0.01, 0.04]	0.01 [0, 0.01]	0.02 [0.01, 0.03]	0.01 [0.01, 0.04]	0.01 [0, 0.02]
**A036**	0.15 [0.05-0.4]	0.16 [0.06-0.49]	0.13 [0.04-0.34]	0.61 [0.23, 2.58]	0.45 [0.14, 0.72]	0.39 [0.13, 0.55]	0.51 [0.21, 1.5]	0.9 [0.14, 1.7]	0.26 [0.1, 0.48]
**A037**	0 [0-0.02]	0 [0-0.02]	0 [0-0.01]	0.01 [0.01, 0.03]	0.01 [0, 0.02]	0.01 [0, 0.02]	0.02 [0.01, 0.1]	0.02 [0.01, 0.08]	0.01 [0, 0.05]
**A038**	0.09 [0.04-0.31]	0.17 [0.05-0.41]	0.15 [0.05-0.4]	1.08 [0.77, 3.43]	2.71 [1.48, 6.86]	2.01 [0.97, 2.92]	1.05 [0.42, 2.99]	2.56 [0.87, 9.58]	1.75 [0.33, 5.51]
**A039**	0.01 [0-0.05]	0.01 [0-0.03]	0.01 [0-0.06]	0.02 [0.02, 0.04]	0.13 [0.02, 0.28]	0.03 [0.02, 0.17]	0.03 [0.01, 0.11]	0.09 [0.02, 0.2]	0.02 [0.01, 0.13]
**A040**	374.84 [265.25-623.91]	322.86 [204.06-659.41]	394.7 [285.2-577.26]	1050.7 [604.08, 1963.57]	1541.41 [1107.58, 2075.36]	1279.46 [704.46, 1535.36]	1036.37 [592.3, 1478.9]	1213.83 [548.78, 1876.52]	782.47 [442.28, 1993.06]
**A041**	108.54 [68.29-187.48]	88.34 [57-209.67]	109.35 [78.29-172.26]	400.73 [172.84, 903.15]	574.74 [376.78, 883.04]	513.07 [242.76, 636.02]	385.57 [179.17, 604.52]	448.33 [196.92, 749.56]	268.42 [147.39, 927.67]
**A042**	0.01 [0-0.05]	0.03 [0.01-0.07]	0.03 [0.01-0.07]	0.03 [0.02, 0.04]	0.01 [0.01, 0.02]	0.02 [0.02, 0.05]	0.05 [0.02, 0.23]	0.02 [0.01, 0.04]	0.01 [0, 0.05]
**A043**	0.32 [0.19-0.7]	0.4 [0.14-0.79]	0.37 [0.18-0.67]	1.57 [0.35, 2.94]	2.16 [1.09, 2.73]	1.28 [0.45, 2.19]	0.94 [0.55, 1.59]	0.58 [0.43, 1.71]	0.66 [0.32, 2.92]
**A044**	28.89 [16.66-46.52]	22.54 [12.46-49.44]	28.67 [15.67-48.7]	127.4 [23.16, 263.19]	116.23 [62.44, 179.29]	76 [63.22, 219.63]	139.39 [32.5, 242.27]	85.65 [27.81, 166.76]	55.27 [21.54, 175.22]
**A045**	30 [19.08-54.37]	24.28 [12.41-57.22]	32.74 [15.99-53.9]	130.41 [51.86, 499.7]	172.89 [104.83, 341.75]	125.87 [52.51, 204.86]	90.75 [43.8, 193.75]	132.17 [43.65, 230.96]	68.95 [31.5, 256]
**A046**	2.38 [1.25-4.13]	1.52 [0.75-3.18]	2 [1.02-2.74]	9.13 [1.36, 20.14]	3.83 [1.73, 6.57]	3.28 [1.17, 5.18]	5.09 [2.05, 10.2]	2.56 [0.61, 6.22]	1.05 [0.65, 7.5]
**A047**	26.12 [17.75-49.17]	25.27 [12.65-50.53]	29.05 [18.16-45.77]	81.45 [25.16, 266.93]	87.68 [14.28, 182.32]	86.18 [47.96, 148.3]	55.54 [33.37, 95.93]	68.25 [45.4, 164.87]	25.7 [9.01, 62.04]
**A048**	0.91 [0.48-1.68]	0.92 [0.36-1.6]	1.13 [0.59-1.67]	11.7 [2.92, 32.41]	9.52 [3.64, 35.52]	6.19 [1.79, 11.27]	8.64 [2.81, 21.12]	14.44 [3.52, 37.62]	2.82 [0.67, 6.51]
**A049**	2.45 [1.64-5.01]	2.92 [1.27-4.88]	2.48 [1.55-4.27]	8.24 [3.15, 36.82]	11.36 [7.53, 25.66]	11.35 [6.51, 16.31]	9.36 [4, 24.28]	5.29 [3.3, 20.79]	7.99 [3.8, 26.41]
**A050**	0.43 [0.2-1.11]	0.62 [0.26-1.21]	0.82 [0.35-1.36]	3.89 [1.09, 15.08]	4.4 [1.26, 9.46]	2.13 [1.01, 4.37]	3.77 [1.6, 5.34]	3.11 [1.52, 9.24]	0.7 [0.4, 3.86]
**A051**	54.18 [26.07-99.14]	46.77 [21.95-90.73]	42.29 [25.09-75.25]	284.26 [78.48, 984.52]	390.29 [38.25, 621.4]	289.93 [102.47, 373.1]	223.16 [50.94, 516.77]	158.01 [54.89, 473.47]	61.27 [19.8, 240.63]
**A052**	1.85 [0.84-3.81]	1.41 [0.72-2.99]	1.56 [0.74-3.64]	22.41 [6.5, 81.09]	14.29 [5.23, 44.34]	14.8 [3.84, 25.73]	17.42 [4.63, 27.86]	6.82 [1.95, 23.66]	8.29 [2.65, 25.62]

**A053**	1.63 [0.9-3.32]	1.45 [0.71-2.45]	1.16 [0.74-2.8]	12.57 [6.54, 63.96]	15.15 [8.44, 28.29]	7.44 [3.78, 11.88]	10.81 [3.14, 36.81]	7.48 [2.05, 23.96]	9.45 [3.46, 28.38]
**A054**	0.19 [0.08-0.38]	0.17 [0.08-0.28]	0.17 [0.07-0.3]	0.74 [0.35, 6.17]	1.43 [0.38, 4.36]	0.76 [0.35, 1.41]	0.8 [0.28, 3.32]	0.57 [0.18, 1.63]	0.5 [0.23, 2.36]
**A055**	0.47 [0.28-1.17]	0.59 [0.21-1.42]	0.69 [0.25-1.36]	4.55 [1.75, 21.76]	4.29 [2.3, 10.04]	3.79 [1.55, 9.95]	6.52 [1.14, 13.35]	2.06 [0.72, 6.04]	1.66 [0.66, 8.49]
**A056**	0.71 [0.41-1.58]	0.68 [0.39-1.47]	0.81 [0.45-1.55]	3.17 [0.37, 15.57]	4.34 [0.57, 7.55]	4.83 [1.9, 8.73]	3.38 [1.21, 8.17]	3.11 [1.47, 7.5]	0.83 [0.16, 4.15]
**A057**	0.13 [0.05-0.3]	0.23 [0.08-0.6]	0.27 [0.11-0.54]	0.1 [0.03, 0.7]	0.23 [0.02, 0.32]	0.19 [0.03, 0.65]	0.25 [0.07, 0.77]	0.18 [0.08, 0.47]	0.13 [0.02, 0.31]
**A058**	0.03 [0.01-0.09]	0.03 [0.01-0.08]	0.03 [0.01-0.08]	0.04 [0.02, 0.16]	0.03 [0.01, 0.07]	0.03 [0.01, 0.05]	0.06 [0.03, 0.15]	0.04 [0.02, 0.12]	0.03 [0.01, 0.09]
**A059**	8.42 [4.4-14.88]	4.79 [1.16-8.29]	3.51 [1.61-6.26]	2.15 [1.06, 4.69]	10.31 [6.51, 18.82]	8.38 [5.6, 14.76]	3.9 [1.96, 9.16]	10.31 [5.34, 18.21]	4.24 [1.42, 9.83]
**A060**	0.01 [0-0.06]	0.01 [0-0.05]	0.01 [0-0.06]	0.02 [0.01, 0.19]	0.01 [0, 0.04]	0.2 [0.01, 474]	0.08 [0.01, 0.53]	0.02 [0.01, 0.05]	0.02 [0.01, 0.09]
**A061**	0.01 [0-0.04]	0.01 [0-0.04]	0.01 [0.01-0.02]	0.05 [0.02, 0.37]	0.05 [0.02, 0.11]	0.05 [0.02, 0.15]	0.03 [0.01, 0.06]	0.03 [0.02, 0.05]	0.03 [0.01, 0.08]
**A062**	5.17 [3.09-7.69]	6.82 [2.89-9.31]	6.13 [3.87-10.25]	16.73 [4.64, 24.44]	9.87 [5.28, 12.28]	3.99 [1.26, 8.17]	6.45 [2.64, 16.94]	6.99 [2.8, 12.79]	4.14 [2.16, 13.36]
**A063**	1.44 [0.8-2.35]	1.42 [0.61-2.61]	1.83 [0.85-2.64]	3.66 [0.8, 4.12]	1.46 [0.64, 2.24]	2.63 [1.28, 4.2]	1.95 [0.87, 4.75]	1.6 [0.45, 2.4]	0.67 [0.43, 2.02]
**A064**	0.01 [0-0.04]	0.01 [0-0.02]	0.01 [0-0.03]	0.02 [0.02, 0.05]	0.02 [0.01, 0.02]	0.02 [0.01, 0.47]	0.04 [0.02, 0.27]	0.03 [0.02, 0.16]	0.04 [0.01, 0.06]
**A065**	0.13 [0.06-0.41]	0.05 [0.02-0.21]	0.07 [0.01-0.15]	0.66 [0.16, 2.99]	0.62 [0.36, 2.1]	0.88 [0.38, 1.23]	0.87 [0.17, 2.36]	0.5 [0.22, 1.49]	0.45 [0.12, 1.68]
**A066**	0.02 [0-0.07]	0.02 [0-0.1]	0.03 [0-0.1]	0.44 [0.03, 0.57]	0.17 [0.08, 0.55]	0.16 [0.08, 0.32]	0.07 [0.03, 0.34]	0.19 [0.05, 0.43]	0.24 [0.03, 0.36]
**A067**	0 [0-0.01]	0.01 [0-0.05]	0.01 [0-0.04]	0.03 [0.01, 0.15]	0.01 [0.01, 0.03]	0.02 [0.01, 0.04]	0.01 [0.01, 0.06]	0.04 [0.01, 0.12]	0.01 [0, 0.04]
**A068**	0.01 [0-0.02]	0.02 [0-0.05]	0.01 [0-0.03]	0.12 [0.02, 0.51]	0.13 [0.03, 0.37]	0.02 [0.01, 0.16]	0.06 [0.01, 0.16]	0.11 [0.02, 0.35]	0.07 [0.01, 0.41]
**A069**	0.69 [0.37-1.3]	0.39 [0.13-0.89]	0.35 [0.14-0.74]	3.18 [0.51, 6.92]	0.47 [0.08, 1.3]	0.19 [0.06, 0.42]	1.55 [0.29, 2.98]	0.42 [0.11, 1.32]	0.22 [0.03, 0.75]
**A070**	0.35 [0.13-0.75]	0.2 [0.05-0.58]	0.17 [0.07-0.42]	2.15 [0.23, 3.18]	0.58 [0.28, 0.97]	0.26 [0.1, 0.68]	1.12 [0.32, 1.81]	0.33 [0.15, 0.8]	0.29 [0.09, 0.63]
**A071**	0.4 [0.17-0.73]	0.73 [0.26-1.49]	0.7 [0.36-1.14]	2.3 [0.67, 5.11]	2.31 [1.08, 3.84]	0.68 [0.41, 1.3]	3.09 [1.52, 6.3]	1.68 [0.69, 3.46]	0.75 [0.3, 1.5]
**A072**	1.05 [0.73-1.95]	1.61 [0.82-2.96]	2.16 [1-2.99]	5 [1.62, 11.65]	2.09 [1.41, 4.15]	1.15 [0.72, 4.06]	3.46 [1.58, 6.7]	2.75 [0.86, 5.06]	0.76 [0.36, 1.62]
**A073**	0.01 [0-0.05]	0.01 [0-0.04]	0.02 [0.01-0.06]	0.05 [0.01, 0.14]	0.02 [0.01, 0.18]	0.03 [0.01, 0.08]	0.05 [0.02, 0.13]	0.03 [0.02, 0.1]	0.03 [0.02, 0.12]
**A074**	0.46 [0.22-0.81]	0.96 [0.45-1.67]	1.14 [0.45-1.78]	4.14 [1.97, 8.08]	1.37 [0.48, 2.63]	0.53 [0.42, 0.89]	2.49 [0.88, 5.44]	1.77 [0.61, 3.51]	0.42 [0.17, 1.6]
**A075**	2.36 [1.16-2.83]	1.74 [1.01-3.74]	1.8 [0.88-3.06]	10.67 [2.97, 25.66]	12.02 [5.85, 28.42]	8.11 [3.64, 15.22]	6.02 [3.07, 10.83]	8.35 [1.88, 23.4]	6.16 [1.92, 18.47]
**A076**	0.01 [0-0.06]	0.01 [0-0.06]	0.01 [0.01-0.04]	0.04 [0.02, 0.22]	0.02 [0.01, 0.08]	0.06 [0.02, 0.1]	0.06 [0.02, 0.41]	0.02 [0.01, 0.11]	0.04 [0.02, 0.1]
**A077**	3.17 [1.72-5.79]	2.93 [1.26-5.73]	3.03 [1.75-5]	10.6 [5.54, 38.18]	14 [7.09, 34.28]	12.57 [4.85, 17.94]	7.6 [3.44, 17.9]	7.63 [3.97, 34.33]	6.27 [2.63, 21.35]
**A078**	0.01 [0-0.03]	0.01 [0-0.02]	0.01 [0-0.06]	0.02 [0.01, 0.21]	0.07 [0.01, 0.23]	0.01 [0, 0.03]	0.1 [0.02, 0.34]	0.02 [0.01, 0.1]	0.04 [0.02, 0.25]
**A079**	1.46 [0.9-3.04]	1.29 [0.7-3.52]	1.54 [0.8-2.67]	5.61 [1.39, 13.91]	5.61 [2.79, 9.37]	4.51 [2.13, 7.17]	4.08 [2.32, 8.12]	5.1 [1.59, 7.42]	2.78 [1.94, 9.23]
**A080**	1.77 [0.94-3.65]	1.53 [0.65-3.44]	1.55 [0.57-2.84]	17.8 [6.48, 53.69]	15.86 [8.43, 34.68]	12.64 [4.65, 18.71]	12.24 [4.48, 45.4]	8.98 [2.18, 31.63]	8.98 [3.08, 33.07]

**A081**	0.06 [0.03-0.17]	0.09 [0.03-0.25]	0.1 [0.03-0.21]	0.65 [0.37, 1.9]	0.54 [0.06, 1.29]	0.41 [0.2, 0.8]	0.52 [0.22, 1.71]	0.69 [0.29, 1.22]	0.18 [0.06, 0.36]
**A082**	0.01 [0-0.03]	0.02 [0.01-0.04]	0.02 [0.01-0.07]	0.06 [0.03, 0.28]	0.07 [0.02, 0.16]	0.03 [0.01, 0.06]	0.07 [0.03, 0.14]	0.03 [0.02, 0.08]	0.04 [0.01, 0.12]
**A083**	0.9 [0.45-1.98]	0.89 [0.3-1.65]	0.76 [0.43-1.54]	9.77 [2.77, 52.77]	7.76 [1.49, 23.73]	7.76 [3.81, 15.32]	9.01 [2.02, 19.56]	2.84 [1.33, 18.56]	1.65 [0.79, 8.8]
**A084**	0 [0-0.01]	0 [0-0]	0 [0-0]	0.01 [0.01, 0.02]	0.01 [0.01, 0.03]	0.01 [0, 0.01]	0.02 [0.01, 0.15]	0.01 [0, 0.01]	0.01 [0, 0.01]
**A085**	0.01 [0-0.03]	0.01 [0.01-0.05]	0.01 [0.01-0.05]	0.05 [0.02, 0.28]	0.1 [0.02, 0.14]	0.05 [0.02, 0.11]	0.08 [0.03, 0.19]	0.04 [0.03, 0.23]	0.02 [0.01, 0.11]
**A086**	0.72 [0.4-1.19]	0.68 [0.24-1.21]	0.77 [0.29-1.37]	4.89 [2, 25.17]	4.54 [1.97, 7.98]	5.3 [1.32, 11.61]	3.45 [1.17, 12.05]	4.71 [0.77, 7.99]	1.5 [0.57, 6.71]
**A087**	0.01 [0-0.05]	0.01 [0-0.03]	0.01 [0-0.05]	0.55 [0.25, 2.33]	0.53 [0.12, 1.04]	0.11 [0.06, 0.17]	0.38 [0.18, 0.68]	0.38 [0.11, 0.77]	0.07 [0.02, 0.5]
**A088**	3.61 [2.15-6.63]	7.23 [3.59-13.75]	8.08 [4.13-14.61]	4.75 [2.53, 10.85]	2.69 [1.18, 4.75]	2.92 [1.67, 12.96]	5.49 [3.19, 11.4]	3.09 [1.9, 4.74]	2.88 [1.86, 5.28]
**A089**	3.28 [2.06-6.03]	5.82 [3.26-12.28]	6.89 [4.15-12.95]	6.51 [2.93, 10.86]	1.95 [1.13, 3.63]	2.77 [1.66, 10.28]	4.84 [3.92, 10.97]	2.67 [1.82, 5.07]	2.85 [1.99, 6.04]
**A090**	0.01 [0-0.04]	0.02 [0.01-0.07]	0.03 [0.01-0.08]	0.54 [0.03, 0.82]	0.2 [0.04, 0.64]	0.12 [0.04, 0.42]	0.1 [0.03, 0.29]	0.11 [0.07, 0.33]	0.04 [0.01, 0.13]
**A091**	0.01 [0-0.02]	0.01 [0-0.04]	0.01 [0-0.04]	0.17 [0.03, 0.53]	0.07 [0.02, 0.45]	0.12 [0.03, 0.27]	0.13 [0.07, 0.4]	0.03 [0.02, 0.32]	0.06 [0.03, 0.23]
**A092**	0.01 [0-0.04]	0.01 [0-0.02]	0.01 [0-0.03]	0.08 [0.03, 0.4]	0.11 [0.02, 0.22]	0.03 [0.02, 0.2]	0.04 [0.02, 0.21]	0.02 [0.02, 0.12]	0.04 [0.01, 0.26]
**A093**	0 [0-0.01]	0.01 [0-0.02]	0.01 [0-0.03]	0.03 [0.02, 0.09]	0.02 [0.01, 0.03]	0 [0, 0.01]	0.02 [0.01, 0.05]	0.01 [0.01, 0.03]	0.02 [0.01, 0.03]
**A094**	0 [0-0.01]	0.01 [0-0.03]	0 [0-0.01]	0.02 [0.02, 0.35]	0.02 [0.01, 0.07]	0.02 [0, 0.06]	0.02 [0.01, 0.06]	0.02 [0.01, 0.07]	0.01 [0.01, 0.06]
**A095**	0.19 [0.11-0.3]	0.09 [0.03-0.19]	0.1 [0.04-0.2]	1.93 [0.7, 4.18]	1.16 [0.11, 1.69]	0.25 [0.09, 0.59]	0.85 [0.19, 2.83]	0.33 [0.13, 1.54]	0.37 [0.04, 1.59]
**A096**	0.21 [0.04-0.49]	0.25 [0.04-1.16]	0.5 [0.04-0.81]	0.04 [0.02, 0.31]	0.02 [0.01, 0.07]	0.05 [0.01, 0.19]	0.07 [0.02, 0.21]	0.05 [0.02, 0.23]	0.04 [0.01, 0.22]
**A097**	0.32 [0.12-0.65]	0.25 [0.07-0.59]	0.36 [0.13-0.74]	8.87 [3.44, 15.66]	3.6 [1.59, 10.01]	1.79 [0.45, 6.21]	1.96 [0.61, 5.12]	1.56 [0.19, 7.58]	1.15 [0.22, 5.62]
**A098**	0.36 [0.16-1.34]	0.27 [0.08-1.06]	0.18 [0.05-0.61]	1.73 [0.24, 5.17]	2.45 [0.48, 3.89]	0.65 [0.33, 1.35]	0.51 [0.21, 1.27]	0.64 [0.2, 4.01]	0.54 [0.12, 2.71]
**A099**	1.88 [1.13-3.17]	1.84 [0.99-3.34]	1.89 [1.17-3.18]	10.07 [4.16, 22.67]	3.73 [1.01, 19.35]	7.88 [2.32, 20.48]	1.57 [0.74, 15.26]	2.34 [0.81, 10.41]	2.86 [0.97, 10.35]
**A100**	0.12 [0.05-0.21]	0.07 [0.02-0.24]	0.04 [0.01-0.16]	0.67 [0.13, 1.5]	0.42 [0.05, 1.07]	0.35 [0.15, 2.15]	0.1 [0.04, 0.82]	0.23 [0.03, 0.86]	0.16 [0.07, 0.32]
**A101**	0.38 [0.18-0.78]	0.52 [0.24-0.85]	0.59 [0.27-1.11]	6.08 [2.16, 11.3]	2.3 [0.75, 6.69]	3.29 [1.11, 5.96]	2.55 [0.55, 5.53]	3.05 [0.77, 3.95]	1.02 [0.42, 9.41]
**A102**	0.91 [0.6-1.72]	1.3 [0.66-2.5]	1.32 [0.74-2.17]	5.67 [1.61, 10.64]	2.51 [1.53, 6.5]	3.84 [0.95, 8.83]	4.78 [0.56, 11.34]	2.53 [1.35, 4.45]	1.97 [1.23, 11.43]
**A103**	0.05 [0.01-0.12]	0.02 [0.01-0.04]	0.02 [0.01-0.1]	0.49 [0.08, 1.5]	0.32 [0.12, 0.85]	0.09 [0.04, 0.33]	0.24 [0.03, 1.5]	0.25 [0.04, 0.62]	0.09 [0.02, 0.41]
**A104**	0.55 [0.31-1.09]	0.77 [0.34-1.87]	0.91 [0.52-1.77]	2.04 [0.66, 3.54]	0.73 [0.36, 1.41]	1.07 [0.29, 2.13]	1.5 [0.57, 5.09]	1.5 [0.78, 2.39]	0.66 [0.39, 1.71]
**A105**	0.48 [0.27-1.1]	1.01 [0.39-1.72]	0.86 [0.41-1.63]	1.9 [0.67, 3.03]	0.77 [0.43, 1.77]	0.52 [0.22, 2.85]	0.95 [0.47, 2.61]	1.29 [0.37, 1.9]	0.55 [0.22, 1]

Values inside the bracket are 1st and 3rd quartiles. Median and quartile values in the tables were indicated by multiplying 10^3. Cord blood samples were classified by 3 conditions [i.e., healthy full-term, preterm without BPD ['No BPD'], and preterm with BPD ['BPD']]. Peripheral blood samples were classified by infant age at time of collection [1-week, 1-month, and 36-weeks postmenstrual age]. Timepoints were stratified by BPD status. *Red boxes* highlight BPD levels across the 4 timepoints: cord blood, 1-week, 1-month and 36-weeks PMA.
