# Supplementary material for: Cord Blood Adductomics Reveals Oxidative Stress Exposure Pathways of Bronchopulmonary Dysplasia
Source: Antioxidants (Basel). 2024 Apr 20;13(4):494. doi: 10.3390/antiox13040494 (PMC11047351; doi:10.3390/antiox13040494)
Supplement: Supplementary file 1 [file antioxidants-13-00494-s001.zip › Supplement_Figure S1.pdf]

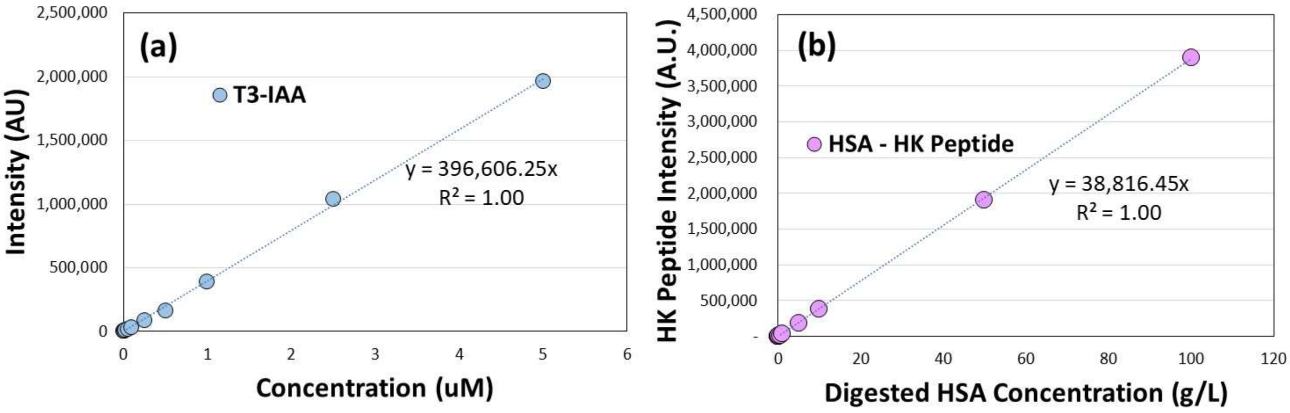

Figure S1. Calibration curves used to estimate adduct concentrations in pmol/mg. Two calibration curves were used to convert adduct concentrations from PAR to pmol/mg. (a) Synthetic T3 peptide was modified with IAA and was used to determine adduct peak area at peptide concentrations ranging from 0.01 to 5 uM. (b) Digested HSA was used to determine HKP peak area at concentrations ranging from 0 to 100 g/L.
